# Electic Journal of Education and Literary Review

**Published:** 1852-03

**Authors:** 


ARTICLE II.

*Electic Journal of Education and Literary Review*, Edited by N. S. Davis, M. D. Corresponding editors, S. S. Randall and O. F. Bartlett.

This work, devoted to the interest of Education in the North West, is issued from the Chicago press monthly at $1,00 per annum. It is printed neatly and edited ably, and should receive the patronage of the public generally.

There is no interest of more vital importance to the future welfare of the profession of medicine, as well as to the community at large, than the proper education of those that are to come after us; for upon it will depend the happiness and usefulness of both.

Physicians generally, are supposed to be well educated themselves, and of course should always take the lead in promoting the cause of education in their respective neighborhoods. Those who do so, will find valuable aid to their labors in the work before us, and will also advance the cause by extending its circulation among their friends and neighbors.

